# Supplementary material for: Reducing the Time-to-Antibiotic by Adapting a Standard of Procedure for the Treatment of Pediatric Cancer Patients Presenting with Febrile Neutropenia—A Comparative Analysis of Two Patient Cohorts
Source: Cancers (Basel). 2025 Oct 10;17(20):3280. doi: 10.3390/cancers17203280 (PMC12564837; doi:10.3390/cancers17203280)
Supplement: Supplementary file 1 [file cancers-17-03280-s001.zip › cancers-3899774-supplementary.pdf]

Supplementary material

SOP algorithm

Families were instructed in the procedural algorithm of FN before first discharge and reminded during each hospital visit. Caregivers regularly measured the patient's auricular or axillar temperature at home, before administration of any antipyretics/analgesics. Upon fulfillment of fever criteria, caregivers were requested to contact the oncology department and to head towards either the pedER or the OD, depending on the time of the day (OD, from Monday to Friday from 8AM to 4PM; pedER, at any other time). Meanwhile, an alarm call was initiated to inform the personnel in the outpatient department (pedER/OD) of the impending arrival of a patient likely to have FN. Thereby enough time was guaranteed to prepare for the admission of the patient and for the diagnostic work-up. An emergency dose of a pre-dissolved broad-spectrum antibiotic (piperacillin-tazobactam) was delivered to the department, where the patient was expected. Upon arrival in the outpatient department (pedER/OD), the patient with suspected FN was evaluated by an experienced medical doctor and a proficient nurse (*is there a life-threatening situation? Is there any sign of sepsis or septic shock?*). Following a thorough body examination aimed to assess the patient's general condition and find an infectious focus, lab works and blood cultures were drawn. After completion of the diagnostic work-up, intravenous antibiotic was administered to the patient without further delay (Fig. 1A).

SOP implementation

Following joint approval by the Department of Pediatric Oncology and the Division of Infectious Diseases, the SOP was published on the hospital's electronic guideline platform next to other SOPs covering any major topic of diagnostic or therapeutic management of pediatric disease. Medical and nursing staff were informed of the update via automatic e-mail and during the regular morning reports. The SOP was then officially presented at a regular hospital-wide routine meeting attended by the entire medical staff. The heads of the pediatric emergency department (pedER) and the outpatient department (OD) subsequently conducted recurring educational and training sessions with the medical teams of their respective units, using previous FN episodes as case scenarios.

The medical team in pedER and OD consists of physicians at all stages of training (residents, fellows, and consultants). In pedER medical doctors rotate daily from other departments to cover emergency shifts. Consequently, physicians who primarily work in the OD are often involved in the initial management of FN episodes in the pedER, ensuring consistency in medical practice across the two departments. In contrast, nursing staff generally remains within their assigned department, with only little exchange between OD and pedER.

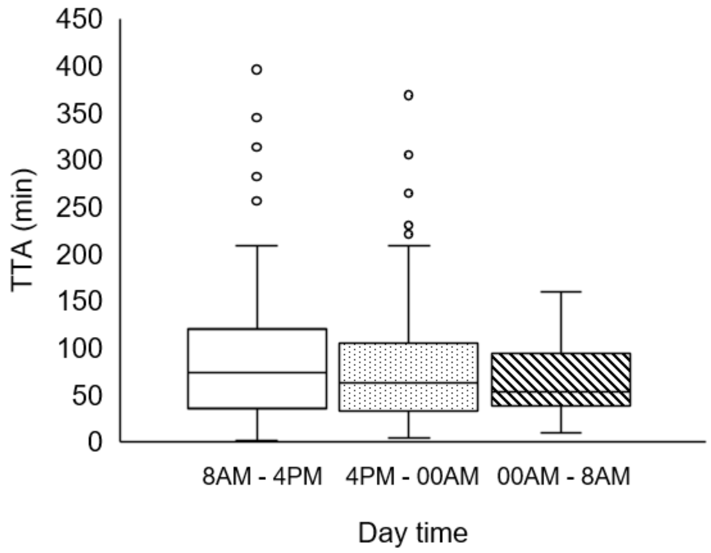

Figure S1. Median TTA similar during different working shifts. 08AM - 04PM median (IQR) = 73 min (35.5 – 119); 04PM - 00AM median (IQR) = 63 min (33 – 102); 00AM - 08AM median (IQR) = 53 min (39 – 89). No statistically significant difference was observed among the three time frames.

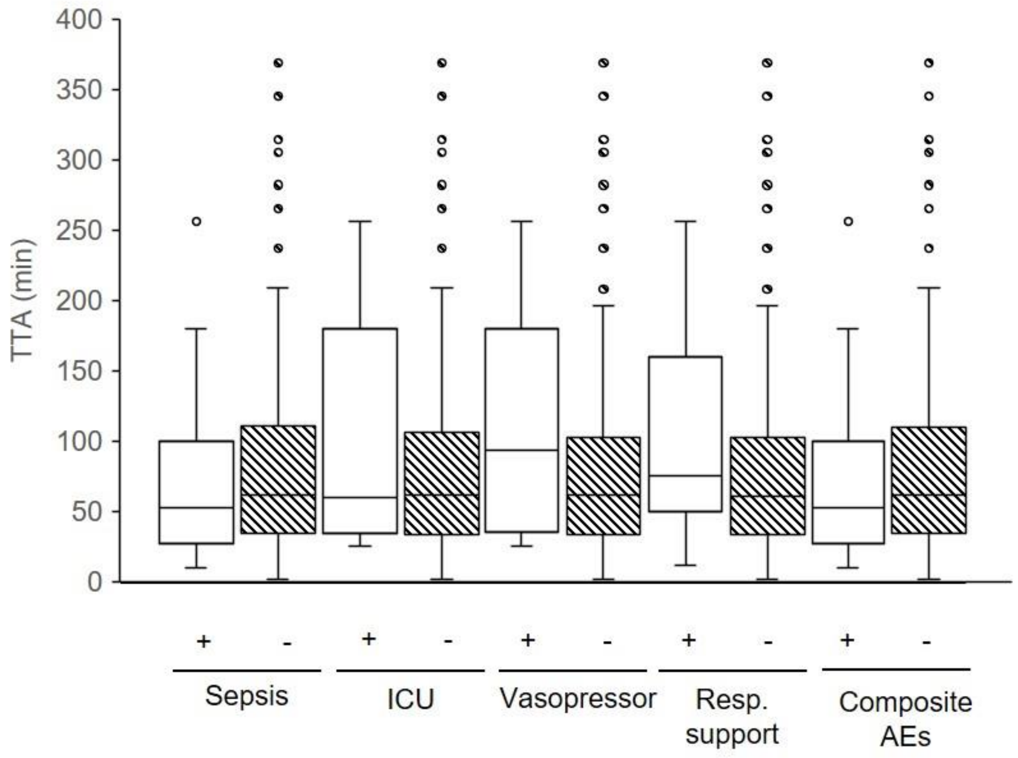

Figure S2. TTA stratified per adverse events. p-value: sepsis 0.49; ICU 0.24 ; vasopressor 0.12; respiratory support 0.17; composite AEs 0.54. Composite adverse events (AEs) are defined as the occurrence of at least one of the followings: sepsis, admission to ICU, use of vasopressors, need of respiratory support or death.

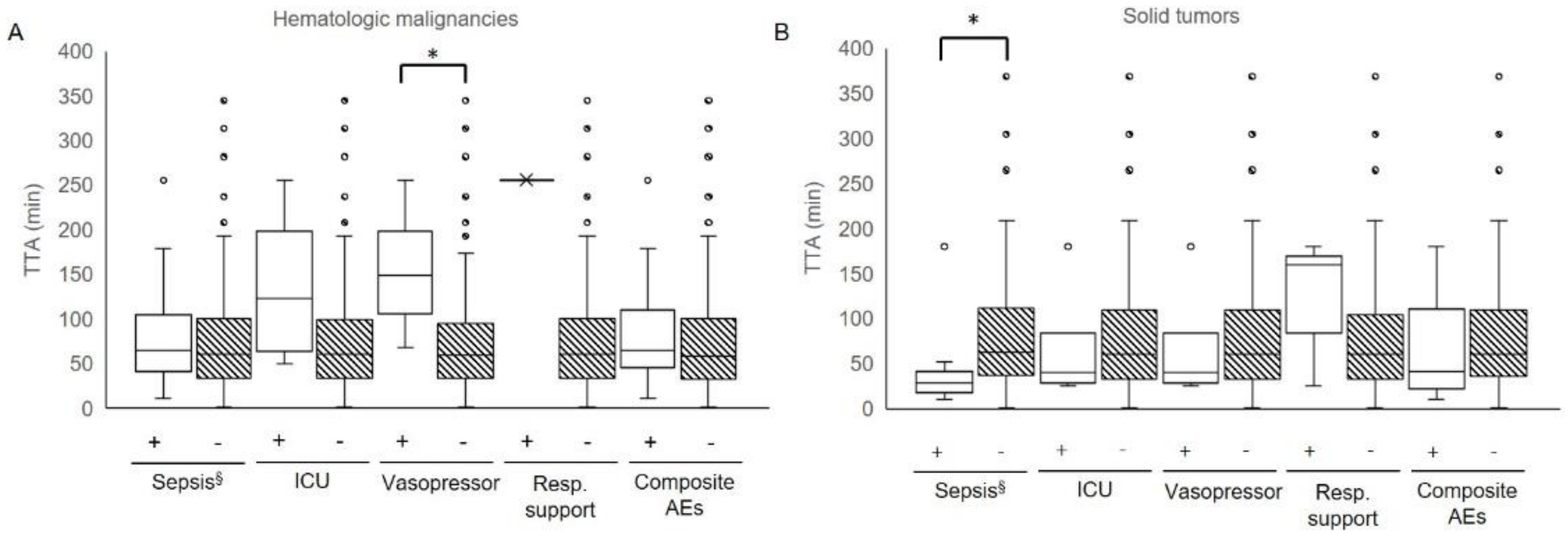

Figure S3. TTA stratified per adverse events in hematologic and solid malignancies. A) Hematologic malignancies, p-value: sepsis 0.793 (N=4); ICU 0.123; vasopressor 0.025 (N=4); resp. support 0.454; composite AEs 0.477. B) Solid tumors, p-value: sepsis 0.046 (N=7); ICU 0.626; vasopressor 0.626; resp. support 0.103; composite AEs 0.411. Composite adverse events (AEs) are defined as in Table S2. Patients with a hematologic malignancy did significantly less often receive vasopressors (median TTA (IQR): "required vasopressors" 149 min (105.5 – 198.3) vs "no vasopressors required" 61 min (34 – 95.3), p < 0.025). Conversely, patients with a solid tumor experiencing sepsis showed a shorter TTA compared to those not developing sepsis (median TTA (IQR): "with sepsis" 29 min (17.5 – 41) vs "without sepsis" 63 min (36.8 – 112.3), p 0.046).

Table S1. Correlation score between length of stay (LOS) and ANC, thrombocyte count and CRP-value

	Pearson's correlation score	Significance
ANC	0.49	n.s.
Thrombocyte count	0.165	<0.05
CRP (initial)	0.009	n.s.
CRP (maximal)	0.308	<0.001

ANC, absolute neutrophile count; CRP, C-reactive protein; n.s., not significant

Table S2. Relationship between ANC, thrombocyte count, CRP-value and AEs occurred during FN episodes

		ANC [x10e3/µL]	RR (ANC<0,2x10e3/µL)	Thrombocyte[x10e3/µL]	RR (Tc<50x10e3/µL)	CRP (initial) [mg/L]	RR (CRP>50 mg/L)	CRP(maximal) [mg/L]	RR (CRP>90 mg/L)
Sepsis	w/o	0.37 (0.23 – 0.51)	1.50	113.9 (94.1 – 133.7)	2.85	32.2 (26.0 – 38.4)	1.90	41.5 (33.9 – 49.0)	8.84
	with	0.17 (0.08 – 0.34) *	(95%CI 0.59 – 3.83)	51.1 (30.3 – 71.8) **	(95%CI 1.28 – 6.32)	51.1 (23.8 – 78.3)	(95%CI 0.88 – 4.13)	168.4 (117.6 – 219.2) ***	(95%CI 4.23 – 18.5)
ICU	w/o	0.35 (0.22 – 0.48)	1.12	108.7 (90.2 – 127.2)	0.80	32.6 (26.6 – 38.6)	2.42	48.3 (39.8 – 56.7)	34.82
	with	0.21 (0.14 – 0.56	(95%CI 0.23 – 5.43)	64.0 (24.2 – 99.8)	(95%CI 0.19 – 3.28)	79.1 (5.8 – 152.4)	(95%CI 0.77 – 4.98)	255.1 (151.4 – 358.8) ***	(95%CI 4.42 - 274.6)
Vasopressor	w/o	0.35 (0.22 – 0.48)	1.12	108.3 (89.8 – 126.9)	0.45	32.8 (26.8 – 38.9)	2.42	47.7 (39.4 – 55.9)	34.82
	with	0.21 (0.15 – 0.56)	(95%CI 0.23 – 5.43)	72.6 (33.7 – 111.6)	(95%CI 0.09 – 2.16)	72.5 (0.9 – 144.0)	(95%CI 0.77 – 4.98)	271.8 (171.1 – 372.6) ***	(95%CI 4.42 – 274.6)
Respiratory support	w/o	0.36 (0.23 – 0.49)	1.50	111.1 (92.1 – 130.1)	2.68	30.2 (24.9 – 35.4)	2.69	45.8 (37.4 – 54.3)	13.68
	with	0.20 (0.03 – 0.43)	(95%CI 0.44 – 5.14)	49.9 (27.5 – 72.2)	(95%CI 0.09 – 2.16)	91.7 (36.7 – 146.6) *	(95%CI 1.10 – 4.30)	193.0 (128.0 – 258.0) ***	(95%CI 4.60 – 40.7)
Composite AEs	w/o	0.37 (0.23 – 0.52)	1.39	116.3 (96.0 – 136.6)	3.08	29.5 (24.2 – 34.8)	1.75	38.2 (31.5 – 44.9)	7.65
	with	0.19 (0.04 – 0.24)	(95%CI 0.73 – 7.76)	48.5 (31.0 – 66.1) ***	(95%CI 1.54 – 6.17)	64.1 (34.2 – 94.0)	(95%CI 0.90 – 3.43)	164.7 (120.2 – 209.1) ***	(95%CI 4.18 – 14.0)
Bacteremia	w/o	0.37 (0.23 – 0.51)	2.38	112.9 (93.4 – 132.5)	4.56	32.7 (26.5 – 38.9)	1.87	47.3 (37.9 – 56.7)	5.96
	with	0.13 (0.02 – 0.29) *	(95%CI 0.73 – 7.76)	52.3 (25,.2 – 79.4) **	(95%CI 1.74 – 11.92)	48.6 (18.9 – 78.3)	(95%CI 0.82 – 4.35)	132.3 (85.0 – 179.6) ***	(95%CI 2.78 – 12.8)

§composite adverse events (AEs) are defined as the occurrence of at least one of the followings: sepsis, admission to ICU, vasopressor, respiratory support, death. ANC, absolute neutrophile count; CI, confidence interval; CRP, C-reactive protein; ICU, intensive care unit; RR, relative risk; w/o, without. Values are reported as mean (95% confidence interval); * p-value <0.05; ** p-value<0.01; *** p-value<0.001

Pathogens in FN episodes

Pathogens were identified in 30/227 FN episodes (13.2%) (Table S3). Ten Gram-positive (33.3%) and twelve Gram-negative bacteria (40.0%) were isolated in blood cultures. Among them there were six staphylococcal infections (6/10, 60% of Gram-positive bacteremia) and five *Escherichia coli* bacteremia (5/12, 41.6% of Gram-negative blood-borne infections). Moreover, *Clostridioides difficile* was detected in stool samples of two patients and one case of a central line infection with *Staphylococcus hominis*. One co-infection with blood-borne *Aspergillus species* and rhinovirus in throat swab was observed. Overall, in four FN episodes a virus was identified as the sole pathogen (4/30, 13.3%; HSV causing a stomatitis, RSV and SARS-CoV-2 linked to upper airway infection and Influenza B in a patient showing symptoms of a common flue).

Table S3. Isolated pathogens during episodes of febrile neutropenia

Organisms	N
All pathogens	30 (265)
Gram-positive bacteria	13 (30)
Staphylococcus epidermidis	3
Staphylococcus hominis	2
Streptococcus mitis	2
Gordonia species	1
Bacillus cereus	3
Clostridium difficile	2
Gram-negative bacteria	12 (30)
Enterobacter species	1
Escherichia coli	5
Campylobacter jejuni	1
Klebsiella pneumoniae	1
Klebsiella oxytoca	2
Pseudomonas aeruginosa	2
Fungi	1 (30)
Aspergillus species	1
Virus	5 (30)
HSV	1
Rhinovirus	1
RSV	1
SARS-CoV-2	1
Influenza-B-virus	1

Table S4. Morbidity and mortality in hematologic malignancies vs solid tumors

		Hematologic malignancies		Solid tumors		
		N	%	N	%	
LOS (days):	≤ 3	5	4.5	14	12	p<0.05
	4 - 6	38	34.5	63	54	p<0.01
	7 - 9	16	14.5	28	24	n.s.
	> 9	51	46.5	12	10	p<0.0001
		N	%	N	%	
Sepsis		18	16.4	7	6	p<0.05
ICU admission		4	3.6	4	3.4	n.s.
Vasopressors / inotropic agents		4	3.6	4	3.4	n.s.
Respiratory support		10	9.1	5	4.3	n.s.
Composite AEs §		21	19.1	12	10.3	n.s.
Mortality		1	0.9	0	0	n.a.
Bacteremia		17	15.5	5	4.3	p<0.01

§ Composite adverse events (AEs) are defined as the occurrence of at least one of the followings: sepsis, admission to ICU, use of vasopressors, need of respiratory support or death. ICU, intensive care unit; IQR, interquartile range; LOS, length of stay; N, number of FN episodes. n.a., not available; n.s., not significant

Table S5. Stratified analysis in hematologic malignancies

	Pre-SOP (N = 48)		Post-SOP (N = 61)		p-value
TTA (min): median (IQR)	89 (50.8 – 141.8)		49 (28.3 – 72.3)		p<0.0001
LOS (days): median (IQR)	8 (5.0 – 12.3)		10 (6.0 – 13.0)		n.s.
	N	%	N	%	
Sepsis	11	22.9	7	11.5	n.s.
ICU admission	2	4.2	2	3.3	n.s.
Vasopressors / inotropic agents	3	6.3	1	1.6	n.s.
Respiratory support	5	10.4	5	8.2	n.s.
Composite AEs§	17	17.5	16	12.3	n.s.
Mortality	1	2.1	0	0	n.a.
Bacteremia	12	25.0	9	14.8	n.s.

§ Composite adverse events (AEs) are defined as the occurrence of at least one of the followings: sepsis, admission to ICU, use of vasopressors, need of respiratory support or death. ICU, intensive care unit. ICU, intensive care unit; IQR, interquartile range; LOS, length of stay; N, number of FN episodes; n.a., not available; n.s., not significant.

Table S6. Stratified analysis in solid malignancies

	Pre-SOP (N = 49)		Post-SOP (N = 68)		p-value
TTA (min): median (IQR)	102 (81.0 – 144.0)		41 (26.8 – 60.0)		p<0.0001
LOS (days): median (IQR)	5 (4.0 – 7.0)		6 (4.0 – 7.0)		n.s.
	N	%	N	%	
Sepsis	2	4.1	5	7.4	n.s.
ICU admission	1	2.0	3	4.4	n.s.
Vasopressors / inotropic agents	1	2.0	3	4.4	n.s.
Respiratory support	3	6.1	2	2.9	n.s.
Composite AEs§	5	10.2	7	10.3	n.s.
Mortality	0	0	0	0	n.a.
Bacteremia	1	2.0	4	5.9	n.s.

§ Composite adverse events (AEs) are defined as the occurrence of at least one of the followings: sepsis, admission to ICU, use of vasopressors, need of respiratory support or death. ICU, intensive care unit. ICU, intensive care unit; IQR, interquartile range; LOS, length of stay; N, number of FN episodes; n.a., not available; n.s., not significant.
